# Supplementary material for: Displacement, personal loss, and psychological strain among physicians and nurses working in Gaza, 2023–2024
Source: PLOS Glob Public Health. 2025 Sep 10;5(9):e0005094. doi: 10.1371/journal.pgph.0005094 (PMC12422511; doi:10.1371/journal.pgph.0005094)
Supplement: S1 Text — (DOCX) [file pgph.0005094.s001.docx]

**S1 Text - Survey**

1. What is your age range?

18-24

25-34

35-44

45-54

55-64

65 or older

2. Where were you born?

North Gaza Governorate

Gaza City Governorate

Middle Gaza Governorate

Khan-Younis Governorate

Rafah Governorate

West Bank and Jerusalem

I was born outside Palestine. Country_____

2. Where were you living before October 7^th^, 2023?

North Gaza Governorate

Gaza City Governorate

Middle Gaza Governorate

Khan-Younis Governorate

Rafah Governorate

3. Where are you staying right now?

(a) Which area of Gaza:

North Gaza Governorate

Gaza City Governorate

Middle Gaza Governorate

Khan-Younis Governorate

Rafah Governorate

(b) What type of facility:

United Nations Relief and Works Agency (UNRWA) Shelter

Refugee Camp

Mosque or Church

School

With family or friends

At home (I have not been displaced)

I do not leave the hospital

Other___________

4. What kind of health worker are you?

-doctor

-nurse

5. What is your gender?

Male

Female

Other

Prefer Not to say

6. What hospital or clinic were you working in before October 7^th^?

(a) Which area of Gaza:

North Gaza Governorate

Gaza City Governorate

Middle Gaza Governorate

Khan-Younis Governorate

Rafah Governorate

(b) What facility:

Al-Ahli Arab Hospital

Shuhada Al-Aqsa Hospital

Al-Awda Hospital

Al-Dorra Hospital

Al-Shifa Hospital

Al-Rantisi Hospital

Beit Hanoun Hospital

European Gaza Hospital

Indonesian Hospital

Kamal Adwan Hospital

Mohammed Yousef El-Najar Hospital

Nasser Medical Complex

Algerian Military Hospital

Al Naser Hospital for Pediatrics

Al Naser Hospital for Ophthalmology

Al Naser Hospital for Psychiatry

Al-Sadaqa Turkish-Palestinian Hospital

Al-Quds Hospital

Kuwaiti Hospital

Al-Helal Emirati Hospital

An UNRWA Primary Care Center

A Ministry of Health Primary Care Center

Another Facility Not Listed________________

7. What hospital or clinic did you work in most recently?

(a) Which area of Gaza:

North Gaza Governorate

Gaza City Governorate

Middle Gaza Governorate

Khan-Younis Governorate

Rafah Governorate

(b) What facility:

Al-Ahli Arab Hospital

Shuhada Al-Aqsa Hospital

Al-Awda hospital

Al-Dorra Hospital

Al-Shifa Hospital

Al-Rantisi Hospital

Beit Hanoun Hospital

European Gaza Hospital

Indonesian Hospital

Kamal Adwan Hospital

Mohammed Yousef El-Najar Hospital

Nasser Medical Complex

Algerian Military Hospital

Al Naser Hospital for Pediatric

Al Naser Hospital for Ophthalmology

Al Naser Hospital for Psychiatry

Al-Sadaqa Turkish-Palestinian Hospital

Al-Quds Hospital

Kuwaiti Hospital

Al-Helal Emirati Hospital

Al Ahli Arab Hospital

An UNRWA Primary Care Center

A Ministry of Health Primary Care Center

Another Facility Not Listed________________

8. Since October 7^th^, did you start working in a different hospital or clinic? Indicate the number of times you have had to change your workplace.

I never changed workplaces

1

2

3

4

More than 4 times

9. Are you still working in a hospital or clinic now?

Yes

no

IF 9=NO: What date did you stop working? _________

IF 9=NO: Why did you stop working?

I was injured

My hospital or clinic shut down and I did not find another to work in

I was forced to evacuate my hospital

People living in my area were forced to evacuate

Concern for my safety or my family’s safety

Another reason _______________

10. Have you provided healthcare services in any setting other than the functioning hospitals and clinics, such as in a shelter or refugee camp?

IF 10=YES

11. Please briefly describe this other service you have provided, if any ______________

12. How else have you changed the way you provide healthcare since October 7^th^, 2023? _________

Sensitive content: The next section includes four “yes” or “no” questions about harm to your family and colleagues. We understand that these experiences may sometimes be difficult to think about, and we appreciate you responding to the best of your ability. You are able to skip any questions if needed.

13. Since October 7^th^, 2023, have any of your family members been killed in an aerial bombardment or as a result of the ground invasion of Gaza?

14. Since October 7^th^, 2023 have any of your family members been seriously injured or disabled in an aerial bombardment or as a result of the ground invasion of Gaza?

15. Since October 7^th^, 2023, have any of your close colleagues been killed in an aerial bombardment or as a result of the ground invasion of Gaza?

16. Since October 7^th^, 2023 have any of your close colleagues been seriously injured or disabled in an aerial bombardment or as a result of the ground invasion of Gaza?

Sensitive content: This section includes is a tool to measure moral distress and secondary trauma which could result from working under the threat of violence. We understand that these experiences may be difficult to think about, and we appreciate you responding to the best of your ability. (ProQOL instrument)

17. Is there anything else you would like your colleagues to know about your experience?

Thank you for completing our survey. If you would like to provide an email so that you can receive summarized results of the study and any resulting future publications, or so that you can be contacted to participate in other future research, please follow this link, where you can provide contact information and indicate reasons you are willing to be contacted. This form to provide contact information is completely separate from the survey. Your survey responses will remain anonymous.

<link to separate form to provide email>
