# Supplementary material for: Displacement, personal loss, and psychological strain among physicians and nurses working in Gaza, 2023–2024
Source: PLOS Glob Public Health. 2025 Sep 10;5(9):e0005094. doi: 10.1371/journal.pgph.0005094 (PMC12422511; doi:10.1371/journal.pgph.0005094)
Supplement: S2 Text — (DOCX) [file pgph.0005094.s002.docx]

**S2 Text – Open Ended Responses:**

**Please briefly describe this other service you have provided, if any (if answered “Yes” to “Have you provided healthcare services in any setting of other than the functioning hospitals and clinics, such as in a shelter or refugee camp?”)**

Dressings

Schools and out points

Medical officer at refugees camp

تقديم الرعاية الطبية العلاج للمرضي في كل الأماكن (*Providing healthcare and medical treatment services everywhere*)

Unrwa shelters

Clinical unit in schools

Primary health care and dressing

In shelters school

I have delivered medical services to displaced individuals at a government school in Rafah, used by the community as a shelter.

Services of primary health care for refugees in the camp, and i also offered my assistance in two places in rafah as an anesthetic resident, but i didn‘t get a place.

Dressing of injured wound

Refugee camp

**How else have you changed the way you provide healthcare since October 7th, 2023?**

i treat patients at home and consult them at the street

At home i treat children

I was the head of NICU dept. at Alshifa hospital where I was responsible for 50 preterm babies throughout the time of war until NOV 10th, when the IDF attacked AlShifa hospital where we were forced to transform the babies to the Emirati hospital via the coordination with the Unicef. Then I moved to the EU hospital in Khan Younis where I currently help my colleagues in the emergency dept in the hospital where we serve an over crowded number of refugees in that area, where most cases suffer from infectious diseases and malnutrition in addition to dealing with baby injuries.

Still more time in the hospital

No big changes

Increased number of incoming cases, especially severely traumatized patients, and working by mass casualty protocols

The increase my skills due to the massive number of cases we are dealing with

Increase the number of cases, especially emergency traumatic services

I graduated from medical school just in 2023, then I started my internship year at August, 2023, completed my obgyn rotation for two months. After October, 7th, I started volunteering in Kuwaiti hospital in rafah as an emergency department dr which was a big new exhausting experience for me.

Emergency care without full history and examination due to huge number and load of refugee

لم أذهب الي العمل لمدة شهر ثم بعد ذلك تطوعت في مكان اخر قريب الي مكان بيتي (*I did not go to work for a month. After that I volunteered in a different place closer to where I live.*)

Provide care for all patients in and out hospital

More difficult to deal with patients, lack of facilities and equipment

We are worked with all people exposed to injury and we provided primary and emergency care

We just provide life saving healthcare

Our care has become rapid and urgent, focused solely on life-saving measures. We are unable to provide patients with their due examinations and comprehensive care due to the overwhelmingly large number of cases.

We became not able to follow systems set by the ministry of health or terms set by the hospital because of the unusual number of people seeking medical care!

I was keeping my efforts as anesthesia doctor or icu doctor , and never changed

There is no adequate medical supplies, so I used alternative improper a medical supplies

Work 24 h by 24h

Not enough time for every patient. Not enough medical supplies. Having to work 24/7 was exhausting so the quality of medical care decreased significantly

**Is there anything else you would like your colleagues to know about your experience?**

Its not our world, our place

My experience getting better with seeing large number of cases in this war

The whole health system in Gaza collapsed, and medical stuff are so exhausted

Is just out of any medical guidelines!

You should do what you are think its right

Be patient with all people especially they exposed the shock of losing family, home and friends, while being exposed to the stress of war

I never imagined I would experience something like this; it's an emotion you can't truly comprehend unless you go through it yourself.

I did not expect to live this stage or to see the amount of victims and injured like what I saw. I was choosing between patients who needed a quick operation, who could endure another time, and for whom we could do nothing. I refused to leave the hospital I was staying. We continued with the treatment, but we reached the point where we were forced to leave our workplace by force of arms and fire. They arrested my colleagues and killed others. I saw death and I thought that doctors were protected.
