# Supplementary material for: Displacement, personal loss, and psychological strain among physicians and nurses working in Gaza, 2023–2024
Source: PLOS Glob Public Health. 2025 Sep 10;5(9):e0005094. doi: 10.1371/journal.pgph.0005094 (PMC12422511; doi:10.1371/journal.pgph.0005094)
Supplement: S1 Table — (DOCX) [file pgph.0005094.s003.docx]

**S1 Table – Individual ProQOL-Health Response Counts, n = 35**

| Question | Never | Rarely | Sometimes | Often | Very Often |
| --- | --- | --- | --- | --- | --- |
| 1. I am happy that I choose to work in healthcare. | 4 | 0 | 9 | 13 | 9 |
| At times I have had to do things that go against my personal values. | 7 | 8 | 16 | 4 | 0 |
| Because of my work, I have unwanted, distressing thoughts. | 3 | 5 | 9 | 11 | 7 |
| I have seen things at work that I believe to be morally wrong. | 3 | 5 | 18 | 4 | 5 |
| I feel supported by my colleagues. | 1 | 4 | 9 | 15 | 6 |
| I feel energized by working with my patients. | 5 | 3 | 6 | 15 | 6 |
| I often find myself thinking about my patients when I am with my family. | 2 | 4 | 9 | 9 | 11 |
| Administrative procedures and rules make my job too hard. | 2 | 7 | 14 | 8 | 4 |
| At times, I have been unable to provide the care that I believe should have been provided. | 3 | 3 | 11 | 15 | 3 |
| I think that I have been affected by the suffering I see at work | 1 | 5 | 7 | 11 | 11 |
| My family supports me in my work in healthcare | 0 | 1 | 7 | 9 | 18 |
| Because of my work, I feel anxious about many things. | 0 | 6 | 8 | 11 | 10 |
| The people who make the decisions that affect my job care about my wellbeing. | 5 | 15 | 10 | 4 | 1 |
| At times, I have felt ashamed of the choices I have made at work. | 7 | 11 | 11 | 5 | 1 |
| I am unhappy at work. | 5 | 9 | 11 | 8 | 2 |
| I feel depressed because of the suffering I see at work. | 2 | 2 | 9 | 14 | 8 |
| I am unhappy because I have observed health workers doing things that I believe are unethical. | 4 | 9 | 12 | 5 | 5 |
| My manager cares about my personal wellbeing. | 10 | 8 | 8 | 6 | 3 |
| My workload seems endless. | 0 | 3 | 7 | 12 | 13 |
| My workplace is an extremely harsh place to work. | 3 | 3 | 8 | 16 | 5 |
| I feel satisfied by my work in healthcare. | 0 | 6 | 11 | 11 | 7 |
| Because of my work, I have very little time for a personal life. | 0 | 3 | 3 | 11 | 18 |
| I have people who I can talk to about my struggles at work. | 0 | 10 | 11 | 9 | 5 |
| I believe I can make a difference through my work in healthcare. | 0 | 3 | 10 | 12 | 10 |
| I have close friends who support me in my work. | 1 | 1 | 10 | 15 | 8 |
| I avoid activities or situations that remind me of patients' suffering. | 1 | 4 | 19 | 8 | 3 |
| I am proud of what I can do to help. | 0 | 4 | 3 | 9 | 19 |
| I feel responsible that I have not always been able to help people. | 0 | 4 | 14 | 10 | 7 |
| My work exhausts me. | 0 | 0 | 7 | 9 | 19 |
| I feel that my work in healthcare makes the world a better place. | 2 | 2 | 7 | 14 | 10 |
