# Supplementary material for: Displacement, personal loss, and psychological strain among physicians and nurses working in Gaza, 2023–2024
Source: PLOS Glob Public Health. 2025 Sep 10;5(9):e0005094. doi: 10.1371/journal.pgph.0005094 (PMC12422511; doi:10.1371/journal.pgph.0005094)
Supplement: S2 Table — (DOCX) [file pgph.0005094.s004.docx]

**S2 Table** – Stratified median (IQR) ProQOL-H scores by gender, profession, most recent workplace governorate, and type of facility lived in. CS – compassion satisfaction, PS – perceived support, BO – burnout, STS – secondary traumatic stress, MD – moral distress

| Gender | CS | PS | BO | STS | MD |
| --- | --- | --- | --- | --- | --- |
| Female (n=10) | 22 (19-22) | 20 (17-23) | 20 (18-21) | 21 (20-24) | 18 (14-19) |
| Male (n=25) | 25 (21-26) | 19 (17-21) | 23 (20-25) | 22 (19-25) | 19 (16-20) |

| Profession | CS | PS | BO | STS | MD |
| --- | --- | --- | --- | --- | --- |
| Doctor (n=25) | 23 (20-24) | 19 (25-21) | 23 (20-26) | 22 (19-25) | 19 (16-20) |
| Nurse (n=10) | 23.5 (21-29) | 21 (18-24) | 20 (18-21) | 21 (18-23) | 18 (15-20) |

| Most Recent Workplace Governorate | CS | PS | BO | STS | MD |
| --- | --- | --- | --- | --- | --- |
| Gaza city (n=4) | 24 (20.5-25) | 18.5 (15.5-20.5) | 23.5 (20.5-25) | 20.5 (19.5-23) | 16.5 (15.5-17.5) |
| Khan younis (n=12) | 22 (20-27.5) | 19 (17.5-22.5) | 20.5 (19-24) | 22 (18.5-25.5) | 19 (17-19.5) |
| Middle gaza (n=3) | 23 (17-30) | 20 (19-21) | 18 (18-26) | 25 (18-27) | 18 (17-19) |
| North gaza (n=1) | **16 (16-16)** | **14 (14-14)** | **22 (22-22)** | **19 (19-19)** | **20 (20-20)** |
| Rafah (n=15) | 23 (20-75) | 21 (17-23) | 21 (20-26) | 21 (20-25) | 19 (14-22) |

| Type of Facility Lived In | CS | PS | BO | STS | MD |
| --- | --- | --- | --- | --- | --- |
| At home (n=8) | 24 (21-28.5) | 21 (19-24) | 20 (19.5-21) | 20.5 (15.5-22.0) | 18 (14.5-19.5) |
| I do not leave the hospital (n=13) | 24 (22-24) | 20 (18-22) | 24 (22-26) | 25 (19-27) | 19 (19-22) |
| Refugee camp (n=3) | 24(18-24) | 21 (16-24) | 23 (19-24) | 24 (21-25) | 17 (15-24) |
| School (n=3) | 23 (19-25) | 20 (17-24) | 18 (17-27) | 21 (18-26) | 18 (10-19) |
| With family or friends (n=8) | 19.5 (17-22.5) | 17 (16.5-19.5) | 21 (19-23.5) | 20.5 (19.5-23.0) | 17 (16-20) |
