# Supplementary material for: Displacement, personal loss, and psychological strain among physicians and nurses working in Gaza, 2023–2024
Source: PLOS Glob Public Health. 2025 Sep 10;5(9):e0005094. doi: 10.1371/journal.pgph.0005094 (PMC12422511; doi:10.1371/journal.pgph.0005094)
Supplement: Attachment — Submitted filename: 20250311 Response to Reviewers (1).docx [file pgph.0005094.s007.docx]

To the editors,

Below please find our responses to each comment. Below each numbered comment is an **Author Response** followed by **Relevant text** excerpts, when applicable.

**Reviewer #1:**

Dear Authors,

Thank you for conducting and writing this important and timely research.
In this paper, the authors try to provide an insight into the psyche of healthcare workers working during a genocide while witnessing horrors and fearing for their lives and their loved ones.
The paper is well written, and the authors use accurate and important framework of necropolitics and settler colonialism.
I am very impressed you were able to conduct such a study in those conditions considering the limited access to health care workers and their constant displacement.
Still, some revisions are required to strengthen the arguments of the article. Kindly see comments below:

1. Lines 194-197 are not clear and can be omitted

**Author response:** Reviewer #2 asked for clarification about language, which we think is important, so we edited this section for clarity:

**Relevant text (lines 249-255):** “We initially intended to use the Arabic version of the survey, however questions 13 and 18 in the Arabic version of ProQOL-H translate “well-being” as “rafahia.” We felt this translation gave these questions a more frivolous connotation with potential to offend participants, given the context. Creators of the instrument require permission to modify the survey, which we were unable to obtain, so we proceeded with the English language version, without modification. English is spoken by the majority of physicians and nurses in Palestine. The ProQOL, another version of the instrument, has also been administered in English to ICU nurses in the West Bank.”

2. Table 1 - please add a column of percentages near the number of participants and a row for “total”.

**Author response**: We implemented the suggested changes.

3. Table 2 – please remove hospitals that have 0 workers in both columns like Al Dorrah and Al Rantisi.

**Author response:** We implemented the suggested changes.

4. This is a useful article for the discussion of “Sumod”
<https://thefunambulist.net/magazine/redefining-our-terms/nakba-sumud-intifada-a-personal-lexicon-of-palestinian-loss-and-resistance>

**Author Response:** Thank you for sharing this piece – we take from it a call to be nuanced and specific in our use of the term, and not to romanticize it. We consider this perspective in revisiting the section in response to your additional comments below as well as those of reviewers 2, 3 (who argued for less emphasis on sumud given that it wasn’t part of the explicit framing of the survey), 4 (who argued that our discussion of sumud, while interesting, is not a conclusion that one can make based on our study), and 5 (who asked for more explanation of the psychological constructs, how they relate to sumud or not, and how they relate to our objective and hypotheses).

**Relevant text (Lines 122-139):** “Many prior studies have measured the psychological burdens carried by healthcare workers in Palestine related to the baseline state of working while subject to Israeli settler-colonialism. Abu-el-Noor et al found that approximately 90% of doctors and nurses surveyed likely had probable posttraumatic stress disorder (PTSD) following Israeli attacks in Gaza in 2014. A follow up study two years later showed minimal changes in reported symptoms, which they attributed to minimal interventions. Veronese et al measured posttraumatic growth and sense of coherence, two related constructs which focus on psychological resources for making meaning out of suffering and mobilizing individual-level coping strategies. Recognizing the many threats to validity of PTSD symptom checklists and other self-reporting instruments in this context, Afana et al described culture-specific expressions for both coping resources and psychological distress, many of which frame Palestinian suffering as it relates to their national cause. Particular attention has been given to the notion of sumud, translated as steadfastness, which is profoundly important in Palestinian society.38 Wick characterizes sumud as a coping mechanism used by Palestinian health workers to navigate the daily challenges associated with their jobs.39 While sumud is indeed an intergenerational mental health resource, it is also fundamentally a cultural and psychological tool to resist colonial oppression.40 As Meari describes, sumud is a practice that allows Palestinian political prisoners to be both victims of colonial oppression and agents who resist that oppression at all costs. The concept of sumud, rooted in the forced displacement of Palestinians since the Nakba of 1947-9, takes on new meaning in the context of the ongoing, accelerated genocide.41-44”

**Lines 365-381:** “For the categories of perceived support and moral distress, existing literature does not have such a faithful basis for comparison. In both of these categories, the vast majority reported scores in an average range. However, all categories can be made more meaningful with the context provided in the literature on Palestinian or Arab idioms of distress and coping strategies. While a mixed-methods study is needed to empirically compare the five ProQOL-H concepts to the idioms of distress or coping resources specific to Palestine, existing literature offers context for the high levels of reported compassion satisfaction. In Arab culture generally, trauma is not necessarily understood as an individual experience, and even the concept of selfhood has a more relational character than in Western societies. Afana et al describe positive religious coping and Palestinian understandings of their liberation struggle as reflections of coping as a structural feature of their social network, rather than merely a quality of individuals. Connection to this structure and the view that exposure to trauma is part of the struggle for national liberation may explain the higher level of compassion satisfaction reported by health workers in Gaza who performed their duties while enduring the existential threat of genocide. These findings and background support the two conceptions of sumud by Wick and Meari – the structured capacity to cope with oppression is also a foundation for anti-colonial resistance. Still, we must interpret these data with caution – interviews and qualitative analysis would be needed to understand if there is meaningful overlap between ProlQOL-H constructs and sumud or other culturally-specific responses to hardship.”

5. The discussion needs to be fleshed out. One of the main findings is that the available questionnaires cannot measure the magnitude of the experience of health workers in Gaza and their comping mechanisms. I understand that it is impossible to conduct larger study at the moment but the authors should point out to the need of conducting open interviews after the genocide is over and synthesize new tools to explore the mental effects of being and treating genocide victims such as grief, bereavement, shock, collective trauma and Sumoud. See for example:
[https://onlinelibrary.wiley.com/doi/abs/10.1002/smi.1429?](https://onlinelibrary.wiley.com/doi/abs/10.1002/smi.1429?casa_token=Fzu6mzNU5pkAAAAA%3ALHS6mCTUXd3K5x34mdRydK3BNMYmY9OgVmeSah0FELVGwQ7NdIE9IY6ROFPEBWKozNPr74x_7isU)

**Author Response:** We have made extensive edits to the discussion section after synthesizing comments from the 5 reviewers. We appreciate the link to this paper as well, which we’ve incorporated into our introduction.

**Relevant text (Lines 178-182):** “Among survivors of the genocide in Rwanda, the peritraumatic response was found to mediate the relationship between the intensity of the traumatic exposure and symptoms of traumatic grief. Their study highlights the importance of measuring psychological experiences proximal to the traumatic exposure and the need to study the specific consequences of genocide as a traumatic exposure, whose nature is highlighted in Albanese’s description.”

6. The authors should discuss that these health workers have probably survived several Israeli wars on Gaza, a fact that perhaps strengthen their coping mechanism. The authors are also expected to discuss briefly the previous studies of Gaza healthcare workers during previous Israeli wars, see the multiple works of Abdelhamid Afana, Guido Veronese and others, they are certainly more relevant than some of the studies mentioned about healthcare workers coping with COVID. see for example:
<https://journals.sagepub.com/doi/abs/10.1177/1359105318785697>
<https://psycnet.apa.org/buy/2020-54569-001>
<https://www.sciencedirect.com/science/article/pii/S0883941717300316>
<https://academic.oup.com/bjsw/article-abstract/43/4/651/1643017>

**Author Response:** We have made extensive edits to the discussion section after synthesizing comments from the 5 reviewers. We agree that these papers you’ve generously linked are more closely relevant and have added them to our introductory framing. We’ve also added an important paper recently published which uses another version of ProQOL in a study of ICU nurses in the West Bank.

**Relevant Text (Lines 122-139):** “Many prior studies have measured the psychological burdens carried by healthcare workers in Palestine related to the baseline state of working while subject to Israeli settler-colonialism. Abu-el-Noor et al found that approximately 90% of doctors and nurses surveyed likely had probable posttraumatic stress disorder (PTSD) following Israeli attacks in Gaza in 2014. A follow up study two years later showed minimal changes in reported symptoms, which they attributed to minimal interventions. Veronese et al measured posttraumatic growth and sense of coherence, two related constructs which focus on psychological resources for making meaning out of suffering and mobilizing individual-level coping strategies. Recognizing the many threats to validity of PTSD symptom checklists and other self-reporting instruments in this context, Afana et al described culture-specific expressions for both coping resources and psychological distress, many of which frame Palestinian suffering as it relates to their national cause. Particular attention has been given to the notion of sumud, translated as steadfastness, which is profoundly important in Palestinian society.38 Wick characterizes sumud as a coping mechanism used by Palestinian health workers to navigate the daily challenges associated with their jobs.39 While sumud is indeed an intergenerational mental health resource, it is also fundamentally a cultural and psychological tool to resist colonial oppression.40 As Meari describes, sumud is a practice that allows Palestinian political prisoners to be both victims of colonial oppression and agents who resist that oppression at all costs. The concept of sumud, rooted in the forced displacement of Palestinians since the Nakba of 1947-9, takes on new meaning in the context of the ongoing, accelerated genocide.41-44”

7. The mentioned healtcare workers, as everyone in Gaza, are also going through a collective trauma that cannot be measured by simply examining multiple individual experiences. It is worth bringing this aspect and the need for further exploration of collective trauma and collective Sumud, see for example:
<https://www.sciencedirect.com/science/article/pii/S2666560324000264>

**Author Response:** Thank you for the reference. This is an important point compatible with other literature on coping among Palestinians and is mentioned in our edited discussion of sumud.

**Relevant text (Lines 371-381):** “In Arab culture generally, trauma is not necessarily understood as an individual experience, and even the concept of selfhood has a more relational character than in Western societies. Afana et al describe positive religious coping and Palestinian understandings of their liberation struggle as reflections of coping as a structural feature of their social network, rather than merely a quality of individuals. Connection to this structure and the view that exposure to trauma is part of the struggle for national liberation may explain the higher level of compassion satisfaction reported by health workers in Gaza who performed their duties while enduring the existential threat of genocide. These findings and background support the two conceptions of sumud by Wick and Meari – the structured capacity to cope with oppression is also a foundation for anti-colonial resistance. Still, we must interpret these data with caution – interviews and qualitative analysis would be needed to understand if there is meaningful overlap between ProlQOL-H constructs and sumud or other culturally-specific responses to hardship.”

8. It is also worth adding to the discussion that the genocide has targeted many hospital and many healthcare workers were killed, abducted and tortured. The fact that these healthcare workers still go to work every day is an act of heroism and resistance that should be saluted.

**Author Response:** We agree that the targeting of healthcare workers for abduction and torture must be noted and did so with edits to our introduction in addition to the discussion where it is already mentioned – we understand it as one of the defining features of the genocide, which is part of the motivation to study and report its effects. The targeting of health workers and the health system has been unprecedented in its magnitude, even in Gaza.

**Relevant Text (Lines 104-118):** “The health system has all but collapsed, with the World Health Organization (WHO) reporting that as of February 14th, 2024, 886 people have been killed in attacks on healthcare since October 2023, while only 18 hospitals in Gaza are partially functional, leaving the remaining 20 hospitals completely out of service. The Palestinian Ministry of Health reported that 986 health workers were killed as of September 2024. Within just the first six weeks of the assault, 25% of hospitals in Gaza had craters left by 2000-lb bombs within lethal range and 83.3% had these craters within infrastructure damage and injury range, and spatial analysis confirms that the destruction of civilian infrastructure including health systems is not random or accidental. This is further supported by documentation of the hundreds of health workers abducted and often tortured or killed in Israeli prisons . Weeks after the withdrawal of Israeli occupying forces from Al-Shifa Hospital following a horrific ground invasion, Palestinian health workers uncovered a mass grave near the hospital containing the remains of patients, some with wound dressings and catheters still in place.32 Shortly thereafter, an even larger mass grave was discovered near Nasser Hospital, with some bodies found with bound hands or feet.33 While not the first instance of attacks on health workers in Palestine, it is unprecedented in its brutality.”

**Reviewer #2**

This is an extremely important paper. Thanks and recognition must in particular be given to the Gaza-based authors and participants who have put in the time and effort to create knowledge despite being subjected to a war of elimination in the ongoing genocide.

The methods are appropriate considering the current circumstances, which lead to the limitations recognized by the authors. I suggest some small modifications that would strengthen the paper and help guide future work on the topic, whether by the same authors or others who wish to conduct work on the same topic.

General note

1. There is obviously a lot to cover in a small amount of space, but considering that the ProQOL includes questions related to the work itself and the work environment in general, it would be important to present (either in the background or the discussion) the potential effect of the siege and targeting of facilities. These have led to serious challenges in carrying out the necessary health work due to destruction or shortage of equipment, disposables, medications, etc. This may affect health workers’ perception of their own work and its limitations (for example, it could on the one hand make them less satisfied with the work because they cannot perform their best, or it could make them more proud because of making the best of a terrible situation).

**Author Response:** In the time we’ve worked on this an important paper on the targeting of healthcare facilities has been published and we cite this as a severe example of destruction of infrastructure. The introduction also incorporates more background on the constructs represented within ProQOL to help make more explicit their relevance and limitations in this context – other reviewers have also requested this.

**Relevant text (98-111):** “The Gaza Strip has endured repeated attacks in recent years resulting in tens of thousands of deaths and injuries, repeated mass population displacement, 1 and the widespread destruction of health services and public health infrastructure.2 Repeated attacks on health workers in Palestine have been documented across many years of Israeli military attacks on Palestine.3-5 Since October 7th 2023, there has been an unprecedented increase in the number and severity of Israeli attacks on the health system in Gaza, resulting in the death and injury of hundreds of health workers, patients and caretakers as well as damage to essential health infrastructure.6 The health system has all but collapsed, with the World Health Organization (WHO) reporting that as of February 14th, 2024, 886 people have been killed in attacks on healthcare since October 2023, while only 18 hospitals in Gaza are partially functional, leaving the remaining 20 hospitals completely out of service. The Palestinian Ministry of Health reported that 986 health workers were killed as of September 2024. Within just the first six weeks of the assault, 25% of hospitals in Gaza had craters left by 2000-lb bombs within lethal range and 83.3% had these craters within infrastructure damage and injury range, and spatial analysis confirms that the destruction of civilian infrastructure including health systems is not random or accidental.”

**Lines 141-160:**

“Psychological Constructs for Health Worker Distress and Well-being

Reflecting health workers’ frequent exposure to trauma22-24 and its consequences for health system functioning,22 the broader psychological literature and health services research on the distress and well-being of health workers is vast. Many related and sometimes overlapping concepts have been developed to describe it, including vicarious traumatization, compassion fatigue, compassion satisfaction, secondary traumatic stress, burnout, perceived support, and moral distress. While there is variation in the definition and application of these concepts, vicarious traumatization generally refers to the stress response of professions with an ongoing empathic relationship with a traumatized client, while secondary traumatic stress (STS) typically refers to an acute stress response which can be experienced by many kinds of health workers when exposed to the suffering of others. Burnout (BO), like STS, is applied to many kinds of health workers but reflects a more gradually lost sense of accomplishment and achievement and an erosion of idealism. Compassion fatigue (CF) and its inverse, compassion satisfaction (CS) reflect a combination of STS and BO, and have also been used to describe the experiences of lay caregivers. Moral distress (MD) is the perception that one’s ethics are compromised due to aspects of their work environment which they cannot control. All of these constructs have been found to play some role in the health worker’s ability to cope with work-related stress. The Professional Quality of Life Scale (ProQOL) incorporates several of these concepts, and was recently administered to Palestinian ICU nurses in the West Bank, with 80% or more reporting average levels of compassion satisfaction, burnout, and secondary traumatic stress. The study team consulted nursing and psychometric experts to approve the tool’s cultural and contextual relevance and construct validity, but did not explicitly explore the political context and its potential bearing on the findings.”

Background

2. Lines 79-80: because the focus of this paper is on health workers, it would be prudent to include additional detail on the toll of Israeli attacks on healthcare workers. For example, you could name the most recent data on health workers killed (total of 986 in the August 2024 report by the Palestinian Ministry of Health) or incarcerated (310 according to the September 25, 2024 update by the Palestinian Government Media Office). You could also include some detail of how and where health workers have been targeted, for example through precision killings whether at work or at home, abductions from workplaces or as they took designated “safe routes”, or as they travelled as part of convoys organized by the WHO and/or ICRC.

**Author Response:** We have included mention of these updated numbers and expand upon the targeting of health workers in the discussion as well – similar emphasis was requested by another reviewer as well. Please see response to comment #8 by Reviewer 1.

3. In addition to this, you can also point to the conclusion reached by many who experienced and/or witnessed the genocide that the destruction of the health sector was a key aim of the Israeli military strategy.

**Author response:** We agree that this is a very key point We note this and also cite more recent papers which empirically demonstrate the intentional targeting of health workers and the health system. For relevant text please see response to comment #8 by Reviewer 1.

Methods
4. The use of an online survey presents limitations, as you discussed, because of the destruction of infrastructure leading to connectivity problems. Did you consider a paper-based survey? Why/why not?

**Author Response:** We sought and acquired approval for the use of paper-based surveys for this reason, however none were distributed. The co-authors limited by connectivity also faced other challenges in recruitment which paper-based surveys could not overcome. We added mention of this.

**Relevant text (384-390):** “Some potentially eligible participants told one recruiter that they did not want to share personal information of any kind because of security concerns and the constant fear of being targeted by the Israeli military. This fear underscores the psychological dimension of the assault. Others simply did not think research was a good use of time when facing such a dire situation. We acquired approval for the use of paper-based surveys however these were not ultimately used, since they require more time to process and did not alleviate security concerns of potential participants.”

5. Lines 149-154: please explain why you chose to include only physicians and nurses at this stage (especially as you note this as a limitation in the discussion)

**Author Response:** Anticipating a small sample, we chose to focus on a more homogeneous subgroup of health workers rather than all health professions in order to improve the validity of our findings for the specific group studied. We note this.

**Relevant Text (Lines 204-208):** “Participants had to be physicians or nurses that worked as employees or volunteers in Gaza before October 7th, 2023 and continued to work there for at least some of the period following. We chose to focus on a more homogenous group of health workers to improve validity since we were expecting a small sample, and we chose to focus on physicians and nurses since they are the majority of frontline health workers in Palestine.”

6. Line 194: “Palestinian members of our team and their” – I think there is a word missing after “their”

**Author response:** We re-wrote this sentence in further explaining our choice of the English-language survey, see below response to comment #7.

7. Lines 194-195: it would be important to specify what exactly you found inappropriate about the questions about health worker treatment by managers in the Arabic version of the ProQOL tool. This would allow for a more open debate about how to modify the Arabic version of the tool in the future.

**Author response:** Questions 13 and 18 in the Arabic version of the questionnaire translate “well-being” as “rafahia.” We felt this translation gave these questions a more frivolous connotation with potential to offend participants, given the context. The Center for Victims of Torture allows for the free use of the survey on the condition that no modifications are made to their instruments. We reached out to them about modifying this but unfortunately received no response. Without permission for a modification, we chose to implement the English version of the survey since we were already considering limiting our sample to doctors and nurses, who generally speak English. We extend the note about language as excerpted below. The limitations of translation here may also be cultural, emphasizing the need for culturally relevant trauma instruments. We further explore this in response to comment #5 from reviewer #1.

**Relevant text (Lines 248-255):** “Among our colleagues, we asked those who would potentially qualify as participants for feedback on the draft survey prior to seeking IRB review. We initially intended to use the Arabic version of the survey, however questions 13 and 18 in the Arabic version of ProQOL-H translate “well-being” as “rafahia.” We felt this translation gave these questions a more frivolous connotation with potential to offend participants, given the context. Creators of the instrument require permission to modify the survey, which we were unable to obtain, so we proceeded with the English language version, without modification. English is spoken by the majority of physicians and nurses in Palestine. The ProQOL, another version of the instrument, has also been administered in English to ICU nurses in the West Bank.”

Discussion
8. Lines 309-310: since several of the authors are health workers from Gaza themselves, this could provide an opportunity to include additional detail on what you interpret to be the potential “cultural differences in the perception of trauma” and “differences in strengths and protective factors”, especially since Gazan health workers are likely to have witnessed previous assaults on Gaza and the pandemic, before the ongoing genocide.

**Author response:** Please see our responses to similar comments made by Reviewer 1, comments #5 through #8.

9. Your interpretation of the findings through the lens of sumud is profoundly important and hopefully the start of further debate. Can your findings tell us more about sumud of health workers in particular? Do you find that your findings align more with Meari (reconciling victimhood and resistance) or Wick (a coping mechanism for health workers) or do they, in the context of a unique experience (a war of elimination, one of the aims of which is to dismantle the health sector as a lifeline for the Palestinian people), open up a potentially novel interpretation of sumud? You don’t have to have the answer, but there is an opportunity for a valuable contribution to the debate (or some new questions), especially when considering, for example, the defiance of Palestinian health workers in the face of threats by the Israeli army and instructions to evacuate, and their insistence to continue operating hospitals as a patriotic and ethical duty.

**Author response:** We greatly appreciate the invitation to expand the conversation about sumud in the context of our data. We balance our desire to respond to these prompts with requests from other reviewers to ensure that our discussion follows more directly from the data we collected. Some of the description of sumud has been moved to the introduction. In our discussion, we interpret the finding of high compassion satisfaction in particular in the context of prior work on coping and well-being among Palestinians. Our perspective is that both Wick and Meari’s conceptions of sumud are compatible with our findings and with the other literature we reference, though more qualitative work is needed to more deeply understand the role of culturally specific experiences of distress and resistance in the consequences of genocide, and hopefully incorporate these into mental health interventions for survivors.

**Relevant text:** Please see our response to comment #4 from Reviewer 1.

10. Although this is not the main aim of the paper, can you speak to the appropriateness of the ProQOL tool itself for the current context of the genocide? Were there areas that needed to be included that were missing? Or others that were included that were not appropriate for the context?

**Author Response:** In the introduction, we add language to define the psychological constructs captured by ProQOL and ProQOL-H. In the thoroughly edited discussion, discussion of sumud and other culture-specific experiences of distress and resistance is accompanied by discussion of the ProQOL-H tool and its limitations. While more work is needed to understand the relevance of constructs in such self-report instruments in the Palestine context, overall the dramatic difference in responses between health workers surveyed during the genocide in Gaza and the ICU nurses in the West Bank surveyed by Ayed et al indicate that responses to the instrument reflect an important aspect of the lived reality in Gaza, albeit incompletely.

**Relevant Text (Lines 141-160):**

**“Psychological Constructs for Health Worker Distress and Well-being**

Reflecting health workers’ frequent exposure to trauma22-24 and its consequences for health system functioning,22 the broader psychological literature and health services research on the distress and well-being of health workers is vast. Many related and sometimes overlapping concepts have been developed to describe it, including vicarious traumatization, compassion fatigue, compassion satisfaction, secondary traumatic stress, burnout, perceived support, and moral distress. While there is variation in the definition and application of these concepts, vicarious traumatization generally refers to the stress response of professions with an ongoing empathic relationship with a traumatized client, while secondary traumatic stress (STS) typically refers to an acute stress response which can be experienced by many kinds of health workers when exposed to the suffering of others. Burnout (BO), like STS, is applied to many kinds of health workers but reflects a more gradually lost sense of accomplishment and achievement and an erosion of idealism. Compassion fatigue (CF) and its inverse, compassion satisfaction (CS) reflect a combination of STS and BO, and have also been used to describe the experiences of lay caregivers. Moral distress (MD) is the perception that one’s ethics are compromised due to aspects of their work environment which they cannot control. All of these constructs have been found to play some role in the health worker’s ability to cope with work-related stress. The Professional Quality of Life Scale (ProQOL) incorporates several of these concepts, and was recently administered to Palestinian ICU nurses in the West Bank, with 80% or more reporting average levels of compassion satisfaction, burnout, and secondary traumatic stress. The study team consulted nursing and psychometric experts to approve the tool’s cultural and contextual relevance and construct validity, but did not explicitly explore the political context and its potential bearing on the findings.”

**Lines 352-364:** “ProQOL-H scores among participants revealed high rates of burnout and secondary traumatic stress alongside very high levels of compassion satisfaction and moderate levels of moral distress. Our analysis of compassion satisfaction, burnout, and secondary traumatic stress benefits greatly from the work of Ayed et al with 162 ICU nurses in the West Bank, where they used ProQOL-5, another version of the instrument which measures those three constructs, and was also administered in English. 37% (13 of 35) of health workers presently surveyed reported high levels of burnout compared to 0% (0 of 162) of West Bank ICU nurses. Secondary traumatic stress was also higher in the present study, with 37% (13 of 35) reporting high levels compared to 0.6% (1 out of 162) of West Bank ICU nurses. Doctors and nurses working during the genocide in Gaza also reported very high levels of compassion satisfaction, 49% (17 of 35) reported high levels compared to 4.9% of West Bank ICU nurses (8 of 162). While nurses in the West Bank suffer from the same regime of apartheid and settler colonialism as health workers in Gaza, the stark difference in reported mental health reflects the reality that the genocidal violence in Gaza is unprecedented, despite many prior assaults by Israeli forces which have also affected health workers.”

11. Based on your findings and interpretation, are you able to suggest future work on this topic, whether that you are planning as a team or that you suggest others to conduct?

**Author Response:** We describe future directions in response to requests from Reviewers 2 and 3.

**Relevant text (393-397):** “While the ProQOL-H instrument offers one account of the psychological toll on health workers in harsh circumstances, the genocide is a distinct traumatic exposure, and qualitative work with survivors is needed to further characterize their experiences and reconcile them with culture-specific idioms of distress and culture-specific coping strategies.”

**Reviewer #3:**

Thank you for writing this important piece.

Abstract:

1. The abstract is well written and clear. I would suggest to also refer to the ICJ and its claim that what we are witnessing is genocide.

**Author response:** We implemented this change, along with other edits to the abstract.

**Relevant text (Lines 40-59):**

“On January 26th 2024, the International Court of Justice (ICJ) recognized plausible grounds for genocide being committed in Gaza by Israel. A hallmark of the violence has been an unprecedented level of targeted attacks on health workers during this period.

To assess the impacts of these attacks, between December 24th 2023 and January 30th 2024 we conducted an anonymous cross-sectional online survey of physicians and nurses working in Gaza prior to and during the genocide. Participants reported on workplace displacement, personal losses, and the psychological impact of working in this context using the Professional Quality of Life Scale for Health Workers (ProQOL-H).

56 participants completed one or more components of the survey. Among 46 participants reporting on displacement, 25 (54%) reported having to change workplaces at least once. Among 41 participants documenting personal loss, 17 (41%) reported that a family member was killed and 32 (78%) reported that a close colleague was killed. Among 35 participants completing the ProQOL-H, 17 (49%) of respondents reported “high” compassion satisfaction (scores of 24-30), 13 (37%) reported high burnout, and 13 (37%) reported high secondary traumatic stress. This is markedly different from CS, BO, and STS scores reported by nurses in the West Bank in a recent study, and likely reflecting the genocide in Gaza.	Further qualitative work with survivors is needed to further characterize their experiences and reconcile them with culture-specific coping strategies such as *sumud*. Culturally relevant, mixed methods follow up to this work is needed to inform interventions to support the recovery of survivors and the rebuilding of Gaza’s healthcare system. This will only be possible when the genocide is brought to a permanent end, along with the conditions which have enabled it.”

2. It would be helpful to start with a brief introduction that outlines the objective of the study, justifies its importance, outlines the research gap, and lists the research questions with which the authors seek to fill the gap. An outline of the article and how it contributes to research and practice would also be helpful. This would help the reader to understand the relevance of the following sections.

**Author response:** We re-drafted parts of the introduction, synthesizing your comments with those of other reviewers, including those asking for certain terms to be defined (settler-colonialism, etc), and open with this requested framing, still beginning with a thorough explanation of the genocide and its context.

**Relevant text (Lines 61-88):** “Israel has a settler-colonial relationship with Palestine and Palestinians, which means that, unlike other colonial regimes focused on exploitation or enslavement, the chief goal is to eliminate the indigenous Palestinians .13 This is partly realized through Israel’s related and widely condemned crime of apartheid, a system of colonial race domination codified in Israeli law which deprives Palestinian people of freedoms and entitlements that are taken for granted as basic human rights in other contexts.13 Daily life under this regime resembles the Jim Crow South. For example, hundreds of thousands of Palestinians in the West Bank and Gaza have been arrested since 1967 and tried in military courts which fail to meet international standards of fair trial, while Jewish settlers living in illegal settlements on occupied territory in the West Bank are exempt from the military orders governing Palestinians. On January 26th 2024, the International Court of Justice (ICJ) recognized plausible grounds for genocide being committed in Gaza by Israel.27 In the report “Anatomy of a Genocide,” the United Nations Special Rapporteur on Palestinian territories occupied since 1967 Francesca Albanese presents the evidence that Israel’s assault on Palestinians in Gaza constitutes genocide, situating these findings in relation to the eliminatory context of settler-colonialism that affects the entirety of occupied Palestine.14 Evidence of genocide presented in the report included details of the following acts: killing members of the group, causing serious bodily or mental harm to groups’ members, and deliberately inflicting on the group conditions of life calculated to bring about its physical destruction in whole or in part, including the destruction of healthcare, food, and sanitation systems. Albanese also presented evidence that these acts were carried out following repeated statements of genocidal intent made by senior Israeli military and government figures. In the words of the Special Rapporteur, “When genocidal intent is so conspicuous, so ostentatious as it is in Gaza, we cannot avert our eyes, we must confront genocide.”15

The targeting of the healthcare system and health workers in Gaza has been a particularly egregious feature of the recent assault. The aim of our study is to learn about the violations suffered by health workers in Gaza during this period and its psychological consequences, putting this in its appropriate political context. Theoretically, we focus on genocide to connect the present experiences of health workers in Gaza with the literature on severe trauma and its consequences, existing literature on culturally specific strategies for coping with trauma in Palestine, and the broader literature on the well-being of health workers and its importance for health system functioning.”

Background:

3. Overall, the background section could be better structured. Right now it is not always easy to follow. My suggestion is:
(a) Background on the current genocidal war on Gaza and connected to this the number of people killed and injured, neighbourhoods flattened, and vital infrastructure destroyed. (Here you can refer immediately to Albanese’s report and the ICJ and other documentation)
(b) Focus on the health system (what used to be there); the almost total destruction of the health infrastructure; and the conditions under which health professionals have to work
(c) The impact of working under such conditions (while living through the genocide themselves) on health workers’ health and mental health as far as we know it
(d) Contextualisation of their experiences in relation of previous onslaughts and what we know about their health and mental health during these. The expected cumulative effect can be mentioned which brings it back to the present.
(e) Final note of why understanding health workers mental health is so important

**Author response:** We took much of your advice and restructured the background section as follows, and included some sub-headings to make the multiple important pieces of context easier to follow.

A) Background on the genocide in Gaza, raising ICJ, Albanese’s report, and related details upfront, including some definitions of settler-colonialism and apartheid requested by other reviewers to better orient readers and define our question. We mention the destruction of the health system upfront because it is both one of the defining features of the genocide and the focus of our study

B) **Targeting of Health Workers and Health Infrastructure in Gaza:** A focus on the destruction of the health system, highlighting the working conditions with special emphasis on the targeting of health workers which has been an exceptional feature compared to prior attacks on Gaza, and among the defining features of this genocide compared to other conflicts

C) **Mental Health in Palestine:** A description of existing literature to on the mental health of Palestinian health workers and its social and political determinants, which includes a highly relevant and recent study using the ProQOL instrument to study the well-being of ICU nurses in the West Bank

D) **Psychological Constructs for Health Worker Distress and Well-Being:** A description of a broader literature on health worker well-being which informed our choice of psychological instrument

E) **Studying the Psychological Effects of Genocide:** An emphasis on the the need to study genocide as a unique traumatic exposure and to do so in its proper context, which sets up our current study which also builds on the findings of the study of ICU nurses which allows for a fruitful comparison. Includes a restatement of the study aim, and a note on our use of the word genocide

Other comments on background:

4. It would be important to state that the health system has been completely destroyed and brough to its knees. More information about the destruction of infrastructure and the difficulty of accessing medication and other vital supplies would provide important contextual insight to better understand the almost impossible situation in which health workers need to carry out their work.

**Author response:** We have done this, responding to related requests for more details about destroyed infrastructure and the targeting of health workers. Please see response to comment #8 by Reviewer 1.

5. The psychological strain among health providers needs to be understood in the wider context of siege, war, and violence. Would it be useful to also refer to other recent onslaughts in 2008/2009, 2012, 2014, and 2021 and then of course the Great March of Return in 2018 (you mention the latter) which caused many injuries and disabilities especially among young people. Health professionals will have seen horrific injuries and other war related health problems before the genocide which leave their psychological marks.

**Author response:** While it is possible that prior exposures to violence may expose the health workers we surveyed to accumulated psychological effects, we cannot infer its influence based on our data. Also, the targeting of health workers in the prior assaults on Gaza was relatively uncommon compared to the systematic, genocidal targeting of health workers and healthcare infrastructure since October of 2023. Because we also have ProQOL scores from this time period from ICU nurses in the West Bank, we focus edits to our discussion on this comparison, which supports our sense that the genocide in Gaza, while part of a continuous historical experience since the Nakba, has been uniquely heinous, and that this is strongly felt by health workers. Understanding the continuity between the genocide and past assaults and the psychological impact of that continuity would be worthwhile, but we feel it is outside the scope of our particular study.

**Relevant text (Lines 344-356):** “ProQOL-H scores among participants revealed high rates of burnout and secondary traumatic stress alongside very high levels of compassion satisfaction and moderate levels of moral distress. Our analysis of compassion satisfaction, burnout, and secondary traumatic stress benefits greatly from the work of Ayed et al with 162 ICU nurses in the West Bank, where they used ProQOL-5, another version of the instrument which measures those three constructs, and was also administered in English. 37% (13 of 35) of health workers presently surveyed reported high levels of burnout compared to 0% (0 of 162) of West Bank ICU nurses. Secondary traumatic stress was also higher in the present study, with 37% (13 of 35) reporting high levels compared to 0.6% (1 out of 162) of West Bank ICU nurses. Doctors and nurses working during the genocide in Gaza also reported very high levels of compassion satisfaction, 49% (17 of 35) reported high levels compared to 4.9% of West Bank ICU nurses (8 of 162). While nurses in the West Bank suffer from the same regime of apartheid and settler colonialism as health workers in Gaza, the stark difference in reported mental health reflects the reality that the genocidal violence in Gaza is unprecedented, despite many prior assaults by Israeli forces which have also affected health workers.”

Methods:

6. The methods section seems to suggest that you didn’t only include Palestinian health workers, but anyone who worked/volunteered in the Gaza health sector before and sometime after October 7th. I think this needs to be made much clearer from the start (i.e., objective and research questions (which are absent). It would also need to feature in the background more strongly. The background needs to highlight that Gaza’s health system is supported by international health workers/volunteers with relevant numerical and demographical information provided.

**Author response:** This is correct. International aid organizations providing healthcare in Gaza include UNRWA most notably, which mostly employs Palestinian people, as they say on their website. Many international health workers supporting the health system do so on a part time basis through temporary missions, but we do not have estimates of how many such individuals are working in the system at any given time. Still, because they are present in some number, likely have close relationships with their Palestinian colleagues, and could also be affected by attacks on healthcare, we did not exclude them in trying to account for the psychological impacts of the genocide. Our study is focused on the impacts of the genocide on health workers in Gaza. Ultimately we only had 5 participants noting a birthplace outside Palestine who may or may not be of Palestinian ancestry. While we were open to the responses of non-Palestinian health workers who work in the system, their experience or its possible difference from the median experience is not our principal focus. Between 91 and 100% of respondents are Palestinian, but because a few participants may not be we say “health workers in Gaza,” in our aims, consistent with our inclusion criteria.

**Relevant text (Lines 83-84):** “The aim of our study is to learn about the violations suffered by health workers in Gaza during this period and its psychological consequences, putting this in its appropriate political context.”

Results:

These are important and well reported.

7. While you moved qualitative quotes in the appendix as there were too few for thematic analysis, I wonder if they could be included in relevant sections as illustrative examples of how participants reflected on what the numbers reveal. You can note the lack of methodological rigour here in the methods section – I just find that these quotes bring the numbers to life and form part of your findings.

**Author response:** Thank you for this suggestion. We agree that the few quotes provided are moving and important, which is why we wanted to make them available. However, we do not present this as a mixed methods study. Instead we mention in our conclusion (and in response to Reviewer 2) the need for a follow up study with rigorous collection of qualitative data to describe the genocide’s psychological impact with richer data, and also inform efforts to better understand and serve those impacted by this genocide and similar forms of political violence.

Discussion:

8. Much of the information here reads like it belongs in the background section. It would have been useful for readers to read about the destruction of hospitals and the mass graves that were uncovered in them beforehand.

**Author response:** Thank you for this suggestion. We have made extensive edits to the discussion section after synthesizing comments from the 5 reviewers. This includes ensuring that more of the discussion follows directly from our results, increased details about health system destruction in the introduction, and the movement of some details (such as those you mention about mass graves) to the introduction. (lines to where we move mass graves mention)

**Relevant text (Lines 93-112):** “Since October 7th 2023, there has been an unprecedented increase in the number and severity of Israeli attacks on the health system in Gaza, resulting in the death and injury of hundreds of health workers, patients and caretakers as well as damage to essential health infrastructure.6 The health system has all but collapsed, with the World Health Organization (WHO) reporting that as of February 14th, 2024, 886 people have been killed in attacks on healthcare since October 2023, while only 18 hospitals in Gaza are partially functional, leaving the remaining 20 hospitals completely out of service. The Palestinian Ministry of Health reported that 986 health workers were killed as of September 2024. Within just the first six weeks of the assault, 25% of hospitals in Gaza had craters left by 2000-lb bombs within lethal range and 83.3% had these craters within infrastructure damage and injury range, and spatial analysis confirms that the destruction of civilian infrastructure including health systems is not random or accidental. This is further supported by documentation of the hundreds of health workers abducted and often tortured or killed in Israeli prisons . Weeks after the withdrawal of Israeli occupying forces from Al-Shifa Hospital following a horrific ground invasion, Palestinian health workers uncovered a mass grave near the hospital containing the remains of patients, some with wound dressings and catheters still in place.32 Shortly thereafter, an even larger mass grave was discovered near Nasser Hospital, with some bodies found with bound hands or feet.33 While not the first instance of attacks on health workers in Palestine, it is unprecedented in its brutality. In November 2023, a Médecins Sans Frontières (MSF) nurse texted his colleagues from the basement of Al-Shifa Hospital, where he and his family were taking shelter during intense Israeli bombardment: “We are being killed here, please do something.”

9. While the reference to sumud is apt, it did not come up in the survey. Conflating sumud with compassion might not be entirely appropriate. It would have been good for the survey to reflect more locally relevant expressions of distress and strengths. This, in my view, is another key limitation: i.e., Western conceptions were inquired into while the interpretation tries to make them locally/culturally relevant without having the necessary data. Considering this, the section on sumud is too detailed and long. Instead, it would have been useful to know how these findings reflect experiences of health workers in other war affected settings. What is particular about this situation, what similarities exist?

**Author response:** We agree with reviewer #3 and #5 that more of the discussion should focus on our results, their limitations, and what they teach us, and this also required more background on the psychological literature behind ProQOL-H. Please see our responses to comments #4 and #6 by Reviewer 1 and comment #10 by Reviewer 2 to see our extensive edits, which maintain the importance and relevance of the literature on *sumud*, but also draw on a broader literature to better explain the relevance and meaning of the ProQOL-H scores.

10. Finally, I would have liked to know what practical lessons the research inspires. What should be done to support health providers in the short-, medium-, and long-term?

**Author Response:** We describe future directions in response to requests from Reviewers 2 and 3.

**Relevant text (393-397):** “While the ProQOL-H instrument offers one account of the psychological toll on health workers in harsh circumstances, the genocide is a distinct traumatic exposure, and qualitative work with survivors is needed to further characterize their experiences and reconcile them with culture-specific idioms of distress and culture-specific coping strategies.”

**Reviewer #4**

Important and meaningful context in para 2, but seems a bit disjointed, especially the reference to food and water deprivation (which seems unrelated to the issue of health care workers—perhaps make more clearly the point that these workers are suffering from the same deprivation and starvation). It appears that the thesis of the paragraph is in the last sentence, so I would rearrange this content by streamlining this and the next paragraph (starting with line 91).

**Author response:** The introduction has been thoroughly restructured and parts of this paragraph (including the part about food and water) have been removed, while other parts are now part of a longer introductory paragraph on the destruction of the health system. Please see our response to comment #8 by Reviewer 3.

2. The context of the Albanese report should lead the following paragraph, as it offers the framing for the rest of the piece. I recognize that genocide is the context in which the workers being studied are living and working under, but the focus of the introduction should be more heavily focused on how the conditions of genocide manifest in these worker’s living and working conditions, and less so on the wider context itself, as to reflect the study.

**Author Response:** As part of a restructuring of the introduction suggested by another reviewer, the general context of the genocide, including reference to the Albanese report, is now the first paragraph of the introduction. The specific argument we are making relates to the need to study genocide as a distinct traumatic exposure, and a defining feature of this genocide has been the unprecedented targeting of health workers with killings, abductions, and torture along with the destruction of health infrastructure. These details have been added to the background, along with more psychological background for ProQOL-H.

**Relevant text:** For an outline of the restructured introduction, please see our response to comment #3 by Reviewer 3.

3. Should there be earlier reference to the ICJ case, and how Albanese’s report offers evidence for the legal definition of genocide? This is not clear in lines 103-107.

**Author Response:** The details are now at the top of the background section.

**Relevant text (Lines 61-88):** “Israel has a settler-colonial relationship with Palestine and Palestinians, which means that, unlike other colonial regimes focused on exploitation or enslavement, the chief goal is to eliminate the indigenous Palestinians .13 This is partly realized through Israel’s related and widely condemned crime of apartheid, a system of colonial race domination codified in Israeli law which deprives Palestinian people of freedoms and entitlements that are taken for granted as basic human rights in other contexts.13 Daily life under this regime resembles the Jim Crow South. For example, hundreds of thousands of Palestinians in the West Bank and Gaza have been arrested since 1967 and tried in military courts which fail to meet international standards of fair trial, while Jewish settlers living in illegal settlements on occupied territory in the West Bank are exempt from the military orders governing Palestinians. On January 26th 2024, the International Court of Justice (ICJ) recognized plausible grounds for genocide being committed in Gaza by Israel.27 In the report “Anatomy of a Genocide,” the United Nations Special Rapporteur on Palestinian territories occupied since 1967 Francesca Albanese presents the evidence that Israel’s assault on Palestinians in Gaza constitutes genocide, situating these findings in relation to the eliminatory context of settler-colonialism that affects the entirety of occupied Palestine.14 Evidence of genocide presented in the report included details of the following acts: killing members of the group, causing serious bodily or mental harm to groups’ members, and deliberately inflicting on the group conditions of life calculated to bring about its physical destruction in whole or in part, including the destruction of healthcare, food, and sanitation systems. Albanese also presented evidence that these acts were carried out following repeated statements of genocidal intent made by senior Israeli military and government figures. In the words of the Special Rapporteur, “When genocidal intent is so conspicuous, so ostentatious as it is in Gaza, we cannot avert our eyes, we must confront genocide.”15

The targeting of the healthcare system and health workers in Gaza has been a particularly egregious feature of the recent assault. The aim of our study is to learn about the violations suffered by health workers in Gaza during this period and its psychological consequences, putting this in its appropriate political context. Theoretically, we focus on genocide to connect the present experiences of health workers in Gaza with the literature on severe trauma and its consequences, existing literature on culturally specific strategies for coping with trauma in Palestine, and the broader literature on the well-being of health workers and its importance for health system functioning.”

4. The framework of settler-colonialism is brought in early in para 4, but requires more explanation for a general public health audience. It seems “apartheid regime” may be more relevant considering the rest of the content in the sentence— otherwise, more explicitly make the connection between genocide and settler colonial elimination for the reader.

**Author Response:** We added a definition of settler-colonialism to help make this connection. Please see our response just above (comment #3 Reviewer 4).

5. The paragraph starting on line 111 seems to be unrelated to the ultimate thesis of the paper. I think you could go directly to line 120 and then into the next para.

**Author response:**

The role of this paragraph is to strengthen the connection between the legal definition of genocide illustrated by Albanese and the empiric study of trauma through the lens of Palestinian mental health and the broader literature on health worker well-being. This calls for attention to the nature of a traumatic exposure – its quality, its severity, and its consequences over the life course. Making this connection in a clear and thorough way is important in conveying why we must not only study the psychological and other consequences of the genocide, but why we must study it *as a genocide*. This exposure is unique not only in its severity but in its quality, for all the reasons that make genocide distinct as a traumatic exposure and also as a legal category. The description by Albanese, which emphasizes a relationship between gruesome violence and egregious violations of dignity, helps to make the case not only for our study but other studies like it. Please see our restructured outline in response to comment #3 by Reviewer 3, and below see the excerpted subheading which as been edited to make these points more explicit.

**Relevant text (Lines 154-189):**

“**Studying the Psychological Effects of Genocide**

Extreme violence against the Palestinian people continues to have a profound psychological impact on health workers in Gaza. For those who survive, they continue to live in perpetual proximity to death.11 The mental health of health workers is important to the functioning of any health system, and must be accounted for when addressing the harm. While the study of nurses in the West Bank in 2024 by Ayed et al reflected an average professional quality of life, the brutal treatment of health workers during the genocide in Gaza needs additional attention.

Genocide is qualitatively distinct as a traumatic exposure and our study of it must take this into account. Alongside the destruction of the healthcare system in the determination of genocide there is also evidence of the destruction of cultural heritage sites, mosques, and churches, which form an important part of the Palestinian social fabric. These components are important in relation to the study of trauma as the nature of traumatic exposure and its context is known to shape psychological sequelae.16-18 In the words of Albanese: “Israeli soldiers have published footage boasting about their killing of families, mothers, children - the bombing of homes, mosques, and schools. Self-incriminating videos show them sadistically mocking and humiliating their Palestinian victims not only by violating their physical integrity and right to life, but also their dignity, their most intimate possessions and spaces that the soldiers have entered and looted, and by desecrating cemeteries and places of worship.”14 Among survivors of the genocide in Rwanda, the peritraumatic response was found to mediate the relationship between the intensity of the traumatic exposure and symptoms of traumatic grief. Their study highlights the importance of measuring psychological experiences proximal to the traumatic exposure and the need to study the specific consequences of genocide as a traumatic exposure, whose nature is highlighted in Albanese’s description.

The psychological effects of any form of extreme trauma on oppressed peoples must take into account the social and political context that shape values, beliefs, and life goals.19 Health workers attacked by the Israeli military during the 2018 Great March of Return, a historic nonviolent uprising in Gaza, pointed to the structural and continuous nature of violence as key features of its impact.20

In order to assess and contextualize the psychological impact of the ongoing genocide on physicians and nurses working in Gaza after October 7th, 2023, we conducted a brief online survey to assess experiences of workplace displacement, personal loss, and the impact of traumatic stress.

We use the word “genocide” to refer to this period of accelerated violence and displacement in Gaza, following preliminary ICJ findings and subsequent detailed reporting by UN Special Rapporteur Albanese.27,14 It is also consistent with more recent reports of the United Nations Special Committee to Investigate Israeli Practices , Human Rights Watch , and Amnesty International all finding that Israel has committed genocide in Gaza. The psychological consequences for health staff enduring this can be understood in relation to a clear understanding of the violent political context, past work on the mental health of Palestinian health workers and other victims of genocide, and the broader literature on the well-being of health workers.”

6. Line 126- some context for Great March of Return needed for those who are not familiar.

**Author Response:** We added some brief background.

**Relevant text (Lines 176-178):** “Health workers attacked by the Israeli military during the 2018 Great March of Return, a historic nonviolent uprising in Gaza, pointed to the structural and continuous nature of violence as key features of its impact.”

7. A stronger case needs to be made in the paragraph leading up to the research statement starting on line 133. I also don’t think enough has been done to bridge the gap between the statement that PTSD and other such measures have been widely critiqued in Palestine and other crisis-affected settings (long before the GMR) and the justification for using similar measures in this study.

**Author response:** PTSD is a particular diagnosis which is quite fraught in a cross-cultural context, including in Palestine. For this reason, we have actually removed the reference to PTSD in this paragraph because we did not administer any diagnostic PTSD screener or similar precisely because of this context. ProQOL-H was chosen because it helps to understand experiences of trauma through people’s relationship with their care-giving work. We’ve added extensive further background about each of the constructs measured by ProQOL-Health. The discussion includes more specific discussion of how these constructs may or may not be relevant in the Palestine context, and what further work would be needed to understand and support these health workers.

**Relevant text:** For relevant text please see our response to comment #10 from Reviewer 2.

8. The recruitment challenges are completely understandable, but can you clarify whether potential participants told study authors the reasons for not participating, or whether these were logical assumptions made by study authors (because this is an important finding in and of itself and is an important comment to the difficulty of doing these work in such settings).

**Author Response:** These were reported by potential participants to one of our co-authors on the ground. We revised the text to make this more clear.

**Relevant text (Lines 376-379):** “Some potentially eligible participants told one recruiter that they did not want to share personal information of any kind because of security concerns and the constant fear of being targeted by the Israeli military. This fear underscores the psychological dimension of the assault.”

9. Interesting that the survey was conducted in English— there is an abundance of literature that demonstrates that part of capturing trauma could include the ability for participants to relay their experiences in their native language, in which different words and concepts may be interpreted differently. Even though “English is spoken by the majority of physicians and nurses in Palestine” as the authors state, could you say more about this decision?

**Author Response:** Please see our response to question #7 from Reviewer 2.

10. The sentence starting on line 194 seems missing a word or two.

**Author Response:** This section was re-written to share more details about our decisions regarding language. Please see our response to question #7 from Reviewer 2.

11. To be clear, were the 79 individuals approached those approached by the seed participants as described earlier? How was it shared with researchers how many were approached in total?

**Author Response:** This was reported in error. We only had a partial accounting of all those invited to take the survey, which was more than 79. We have removed this and also removed the response rate calculated on this basis (which was falsely high).

**Relevant text (Lines 255-257)**: “57 surveys were returned. One response was removed as it was suspected to be a duplicate due to substantial similarity with another response in completion time, demographics, and the formulation of open-ended responses.”

12. Do the recruitment efforts skewing male parallel the overall gender disparity in medical workers in Gaza/Palestine more broadly?

**Author response:** It is true that physicians in particular in Palestine are disproportionately male, with WHO estimates up to 80%. It also likely reflects the fact that most of our recruitment team was male. <https://applications.emro.who.int/docs/WHOEMHRH646E-eng.pdf>

13. Table 2 is very interesting, tracing the displacement of the workers. Would be very useful to see this translated to a line or bar graph (perhaps just the governorates as opposed to the individual hospitals).

**Author response:** To visualize the displacement of workers, with most workers moving from various governorates to Rafah, we added two pie charts to show reported workplaces prior to Oct 7th, 2023 and recent reported workplaces at the time of the survey.

**Figure 1 (Lines 284-286): Workplace displacement reported by doctors and nurses by Gaza Governorate, December 2023 to January 2024 (n = 46)**

14. Concepts like “work-life balance” seem insufficient in the face of what these workers are experiencing. Could you describe how well the ProQOL-Health mapped onto capturing meaning in the lived experience of the participants?

**Author Response:** We’ve devoted more space in the introduction to explaining the constructs captured by ProQOL-H, and more space in the discussion to the relevance and limitations of these constructs.

Relevant text: Please see our response to comment #10 from Reviewer 2.

15. More detail about the ProQOL-Health is needed. Has it been used in other settings of war/genocide? In the discussion, a few studies are addressed, but it’s unclear that the work of nurses in Jordan during COVID bears much relation to the experiences of health workers in Gaza today.

**Author response:** Please see our response to comment #10 from Reviewer 2 regarding ProQOL-H, and please see our response to comment #6 from Reviewer 1 regarding studies of health worker mental health in Gaza.

16. This descriptive analysis would be made significantly more robust with inferential statistics. I also think it would be interesting to see whether any of the demographic characteristics had any bearing on the results, for example by gender, by the type of facility in which they are staying, or by profession. Does that affect their level of burnout or perceived support, for example? The discussion would be greatly strengthened if it depended more on the results presented from the study and explored them.

**Author Response:** Unfortunately, we think the lack of representative sampling and small sample size are the main limitation to our data. To further explore this data, we stratified each ProQOL outcome by gender, by profession, by most recent workplace governorate, and by the type of facility lived in. Tables displaying this have been added to the appendix, and our commentary can be found below:

**Relevant text (Lines 249-251)** “To further explore the ProQOL-H outcomes and generate hypotheses to drive future work, we stratified ProQOL-H outcomes by gender, by profession, by most recent workplace governorate, and by the type of facility lived in.”

**Lines 312-317:** “Stratified cell counts did not suggest major differences in median ProQOL-H scores based on gender or profession. (Appendix Table 3) Respondents living with family or friends (n=8) appeared to have lower compassion satisfaction and perceived support than those still at home, staying in the hospital, or living in a refugee camp or school. The single individual working most recently in North Gaza at the time of the survey reported far lower compassion satisfaction and perceived support than the median values reported from those working in Gaza City, Khan Younis, Middle Gaza, and Rafah.”

**Appendix Table 3** – Stratified median (IQR) ProQOL-H scores by gender, profession, most recent workplace governorate, and type of facility lived in. CS – compassion satisfaction, PS – perceived support, BO – burnout, STS – secondary traumatic stress, MD – moral distress

|  | CS | PS | BO | STS | MD |
| --- | --- | --- | --- | --- | --- |
| Female (n=10) | 22 (19-22) | 20 (17-23) | 20 (18-21) | 21 (20-24) | 18 (14-19) |
| Male (n=25) | 25 (21-26) | 19 (17-21) | 23 (20-25) | 22 (19-25) | 19 (16-20) |

|  | CS | PS | BO | STS | MD |
| --- | --- | --- | --- | --- | --- |
| Doctor (n=25) | 23 (20-24) | 19 (25-21) | 23 (20-26) | 22 (19-25) | 19 (16-20) |
| Nurse (n=10) | 23.5 (21-29) | 21 (18-24) | 20 (18-21) | 21 (18-23) | 18 (15-20) |

|  | CS | PS | BO | STS | MD |
| --- | --- | --- | --- | --- | --- |
| Gaza city (n=4) | 24 (20.5-25) | 18.5 (15.5-20.5) | 23.5 (20.5-25) | 20.5 (19.5-23) | 16.5 (15.5-17.5) |
| Khan younis (n=12) | 22 (20-27.5) | 19 (17.5-22.5) | 20.5 (19-24) | 22 (18.5-25.5) | 19 (17-19.5) |
| Middle gaza (n=3) | 23 (17-30) | 20 (19-21) | 18 (18-26) | 25 (18-27) | 18 (17-19) |
| North gaza (n=1) | 16 (16-16) | 14 (14-14) | 22 (22-22) | 19 (19-19) | 20 (20-20) |
| Rafah (n=15) | 23 (20-75) | 21 (17-23) | 21 (20-26) | 21 (20-25) | 19 (14-22) |

|  | CS | PS | BO | STS | MD |
| --- | --- | --- | --- | --- | --- |
| At home (n=8) | 24 (21-28.5) | 21 (19-24) | 20 (19.5-21) | 20.5 (15.5-22.0) | 18 (14.5-19.5) |
| I do not leave the hospital (n=13) | 24 (22-24) | 20 (18-22) | 24 (22-26) | 25 (19-27) | 19 (19-22) |
| Refugee camp (n=3) | 24(18-24) | 21 (16-24) | 23 (19-24) | 24 (21-25) | 17 (15-24) |
| School (n=3) | 23 (19-25) | 20 (17-24) | 18 (17-27) | 21 (18-26) | 18 (10-19) |
| With family or friends (n=8) | 19.5 (17-22.5) | 17 (16.5-19.5) | 21 (19-23.5) | 20.5 (19.5-23.0) | 17 (16-20) |

17. Disappointing that there were insufficient qualitative responses, as I think this could make the study more robust.

**Author Response:** Please see our response to comment #7 by reviewer #3 for our perspective on the qualitative responses.

18. While I wholeheartedly agree with the second half of the sentence (line 294), I’m not sure how to interpret the beginning : “Capturing the experiences of Palestinian health workers forms part of a wider process of re-humanisation”—to whom are these workers re-humanizing themselves?

**Author Response:** We removed this sentence.

19. The relationship between sumud and moral distress is unclear to me. I assume these workers perceive they are acting with high ethics, but are just limited by circumstance. Thus, it is unclear why they would be assumed to have high moral distress. I have similar questions about the compassion satisfaction measure. I would assume, again, that these workers do feel good about helping their people despite the conditions, and in fact that is what keeps them able to work. Why is the assumption that it would be low? I think it would be useful to define these constructs earlier in the paper and the factors that have been shown to affect them.

**Author response:** This point is well taken – we’ve explained these constructs more thoroughly as described above. Moral distress is not “feeling good about helping people,” this is a bit closer to compassion satisfaction. Moral distress is the feeling of violating one’s own ethics without being in control, and can even include a sense of complicity. Because there is no good empiric basis to map this onto culture-specific concepts like *sumud* in our data or in the literature, we place less emphasis on the relationship between moral distress and *sumud* in the revised introduction and discussion. Please see our responses to comments #4 by Reviewer 1 and #10 by Reviewer 2.

20. I am not sure the framing of “protected by resilience” is sufficiently explained in line 330.

**Author response**: Revised text in the introduction and discussion regarding sumud no longer include this sentence (or any theoretical discussion of resilience as a psychological construct).

Relevant text: Please see our response to comment #4 from Reviewer 1.

Overall, this paper offers a unique insight into healthcare workers that have the unprecedented task of attempting to deliver care, and survive, while surrounded by countless threats. The ability to conduct this study at all at this time shows remarkable commitment, and the perspectives of these workers in such a time are invaluable, including as evidence as is noted in the discussion.

21. However, the introduction needs to be brought together into a cohesive background and justification for the study at hand, including discussing the experiences of these healthcare workers (there are some examples of these, but it needs to be presented more systemically). Have there been other studies of health workers during genocide or other mass atrocities? Are there specific threats genocide and settler colonial elimination pose to the conditions of these workers that are different from the direct and indirect violence of war (which they are also experiencing)?

**Author response:** To improve the total cohesiveness of the manuscript and to ensure that its framing reflects a conversation between the study we conducted and existing literature, the introduction has been restructured – please see our response to comment #3 by Reviewer 3. The discussion of our results focuses on the ProQOL-H outcomes and is more strongly grounded in the theory of the constructs themselves, with reference to relevant literature on genocide as a traumatic exposure and literature on Palestinian mental health. Please see our responses to comment #10 by reviewer #2 and comments #4 and #5 by reviewer #1.

22. Despite the obvious and acceptable limitations of conducting such a study, the data analysis could be more rigorous. The demographic information is not as useful if it is not tied to specific outcomes of the study. Some details of the methodology also need to be more clear, not just for the benefit of this study but for the benefit of other researchers who wish to conduct similar studies under such conditions across contexts. I also think use of the ProQOL-Health tool needs to be better justified, as there appear to be some aspects of it that are less relevant in this setting, and it is not being coupled with qualitative analysis to help describe some of the outcomes.

**Author response:** Thank you for this synthesis – we hope our additional analyses and visualizations, additional details about methods, and revised set up for ProQOL-Health together speak to these valid critiques.

23. I think the displacement angle of the workers is an interesting finding, and should be described and discussed more.

**Author Response:** The most complex aspects of the data and discussion relate to mental health, so we’ve only made a brief addition to discuss these results (along with the new figure created to visualize them):

**Relevant text (lines 331-336):** “In our study of doctors and nurses working during Israel’s genocide against the Palestinian people in Gaza between late December 2023 and January 2024, most respondents reported that they had been displaced from their workplaces. Their experiences of displacement are shared by 1.7 million Palestinians who have been displaced into the southern Rafah governorate.^6^ The repeated displacement into increasingly crowded homestays and workplaces is a condition health workers shared with all people in Gaza, and this is a factor in their reported distress and their responses to that distress.”

24. The discussion should focus more on explaining the results found, and explaining some of the assumptions seemingly made by the authors about what was found in the study. There is a long discussion of sumud and resilience in the discussion that, while interesting, can not be elucidated from the data collected in the study. Perhaps this context would fit better in the introduction to give context as to why certain outcomes would be hypothesized.

**Author response:** Please see our responses to comment #10 by Reviewer 2, comments #4 and #5 by reviewer #1, and to comment #21 by Reviewer 4.

**Reviewer #5**

Thanks for the opportunity to review this paper, which studies the impact of Israel’s genocide in Gaza on healthcare workers in settings across Gaza. The authors have conducted the ProQl-Health survey, and have studied the responses of 56 participants. This is a valuable piece of work in that it provides important information about the effects of the genocide which will be of use to scholars of health and genocide, as well as policy-makers. It is not publishable in its current form, but I am supportive of its eventual publication. However, I have some serious reservations that I think should be addressed in a revised version of this work.

1. There isn’t enough exposition on the political context of this article. While I agree with the ideas expressed in this work, not everyone will, and more explanation is needed. The authors state that Israel is a settler colonial regime, which is obviously true to those of us who have studied this context, but sceptical or ill-informed readers would benefit from a line or two to explain this terminology and give a couple of basic pieces of evidence for it. Similarly, when the authors mention the Great March of Return. This is a global health journal, not a political science one. Readers will need some background.

**Author response:** We have added this additional background with definitions to the top of the introduction, and in the section where the Great March of Return is mentioned.

**Relevant text (Lines 61-88):** “Israel has a settler-colonial relationship with Palestine and Palestinians, which means that, unlike other colonial regimes focused on exploitation or enslavement, the chief goal is to eliminate the indigenous Palestinians .13 This is partly realized through Israel’s related and widely condemned crime of apartheid, a system of colonial race domination codified in Israeli law which deprives Palestinian people of freedoms and entitlements that are taken for granted as basic human rights in other contexts.13 Daily life under this regime resembles the Jim Crow South. For example, hundreds of thousands of Palestinians in the West Bank and Gaza have been arrested since 1967 and tried in military courts which fail to meet international standards of fair trial, while Jewish settlers living in illegal settlements on occupied territory in the West Bank are exempt from the military orders governing Palestinians. On January 26th 2024, the International Court of Justice (ICJ) recognized plausible grounds for genocide being committed in Gaza by Israel.27 In the report “Anatomy of a Genocide,” the United Nations Special Rapporteur on Palestinian territories occupied since 1967 Francesca Albanese presents the evidence that Israel’s assault on Palestinians in Gaza constitutes genocide, situating these findings in relation to the eliminatory context of settler-colonialism that affects the entirety of occupied Palestine.14 Evidence of genocide presented in the report included details of the following acts: killing members of the group, causing serious bodily or mental harm to groups’ members, and deliberately inflicting on the group conditions of life calculated to bring about its physical destruction in whole or in part, including the destruction of healthcare, food, and sanitation systems. Albanese also presented evidence that these acts were carried out following repeated statements of genocidal intent made by senior Israeli military and government figures. In the words of the Special Rapporteur, “When genocidal intent is so conspicuous, so ostentatious as it is in Gaza, we cannot avert our eyes, we must confront genocide.”15

The targeting of the healthcare system and health workers in Gaza has been a particularly egregious feature of the recent assault. The aim of our study is to learn about the violations suffered by health workers in Gaza during this period and its psychological consequences, putting this in its appropriate political context. Theoretically, we focus on genocide to connect the present experiences of health workers in Gaza with the literature on severe trauma and its consequences, existing literature on culturally specific strategies for coping with trauma in Palestine, and the broader literature on the well-being of health workers and its importance for health system functioning.”

**Relevant text (Lines 176-178):** “Health workers attacked by the Israeli military during the 2018 Great March of Return, a historic nonviolent uprising in Gaza, pointed to the structural and continuous nature of violence as key features of its impact.”

2. There also isn’t enough exposition on the psychological details of the article. This is a much more serious concern, because it’s currently preventing me from understanding the authors’ findings. I had to look up “compassion satisfaction” (I was chosen to review this article based on my knowledge of Palestine, not psychology), which shouldn’t be the case for technical words. But more importantly, I don’t understand the authors’ findings. Is the central claim that sumud is protective against the kind of serious moral distress that might be expected of healthworkers in a genocide? If so, say so directly! But also say what kind of expectations you had, and what those expectations are based upon. Have similar studies been done in other conflict situations, and the moral distress was greater? I’m a bit lost as to what is being concluded. At another point, the authors mention that PTSD is not appropriate in relation to the violence experienced by Palestinian people, but they don’t explain it. More generally, the authors need to slow down and give more definitions, more explanations, more real-world examples, and more references.

**Author response:** Please see our responses to comment #10 by reviewer #2 and comments #4 and #5 by reviewer #1.

3. The discussion section in general is quite unclear and needs to be almost entirely rewritten. It starts with a paragraph that is a page long and that is not so much discussion of the data (as it should be) as a political update on the situation. I don’t object to this information being included, but first you should discuss your data! This is the section in which you need to carefully describe your expectations regarding compassion fatigue and carefully explain why you think they aren’t met here.

**Author response:** Both the discussion and introduction have undergone thorough editing, in general guided by the call to ensure the discussion focuses more on the data we collected, and that the introduction provides the necessary context to understand what the data means. Please see our responses to comment #10 by reviewer #2 and comments #4 and #5 by reviewer #1.
