# Supplementary material for: Displacement, personal loss, and psychological strain among physicians and nurses working in Gaza, 2023–2024
Source: PLOS Glob Public Health. 2025 Sep 10;5(9):e0005094. doi: 10.1371/journal.pgph.0005094 (PMC12422511; doi:10.1371/journal.pgph.0005094)
Supplement: Attachment — Submitted filename: 20250515 Response to Reviewers.docx [file pgph.0005094.s008.docx]

**Reviewer #2**

1. Thank you so much for your work on this. It's really an important and excellent paper, and the revisions you have made greatly improve it. Just one small comment:
   Lines 118-127: in addition to the challenges to PTSD frameworks that you identified, it’s also important to mention the debate about whether PTSD can even be applied in the Palestinian (especially Gazan) context, due to the fact the trauma has not ended. See Samah Jabr for example.

**Authors’ Response:** Thank you for your kind review. The debate about PTSD in Palestine is the reason we avoided the many self-report instruments which measure PSTD symptoms in order to screen for the diagnosis.

**Action taken:** We added a reference to a relevant piece by Samah Jabr highlighted below.

**Relevant text (lines 124-127):** “Recognizing the many threats to validity of PTSD symptom checklists and other self-reporting instruments and debate over the validity of PTSD in the Palestinian context(24), Afana et al described culture-specific expressions for both coping resources and psychological distress, many of which frame Palestinian suffering as it relates to their national cause.(25) ‘

**Reviewer #3:**

Thank you for sharing the revised version of this important manuscript. I believe it is nearly there. However, the background and discussion sections would benefit from further streamlining and greater clarity regarding the study’s objective, research questions, and relevant context. As it stands, the reader may struggle to grasp the focus of the article at the outset. That said, the methods section has improved, and the results are both highly informative and important

Abstract:

1. It would have been helpful to know who the participants were—specifically, that they were physicians and nurses from Gaza and international staff working in Gaza.

**Authors’ Response:** We appreciate the reviewer for this comment. A similar comment was made by Reviewer #3 in the original request for revisions. We do not want to emphasize the potential inclusion of a very small number of international staff included in our study – between 91 and 100% of participants were born in Palestine.

Please see also the relevant comment from the previous response to reviewers:

“Reviewer #3 Comment 6: The methods section seems to suggest that you didn’t only include Palestinian health workers, but anyone who worked/volunteered in the Gaza health sector before and sometime after October 7th. I think this needs to be made much clearer from the start (i.e., objective and research questions (which are absent). It would also need to feature in the background more strongly. The background needs to highlight that Gaza’s health system is supported by international health workers/volunteers with relevant numerical and demographical information provided.

**Author response:** This is correct. International aid organizations providing healthcare in Gaza include UNRWA most notably, which mostly employs Palestinian people, as they say on their website. Many international health workers supporting the health system do so on a part time basis through temporary missions, but we do not have estimates of how many such individuals are working in the system at any given time. Still, because they are present in some number, likely have close relationships with their Palestinian colleagues, and could also be affected by attacks on healthcare, we did not exclude them in trying to account for the psychological impacts of the genocide. Our study is focused on the impacts of the genocide on health workers in Gaza. Ultimately we only had 5 participants noting a birthplace outside Palestine who may or may not be of Palestinian ancestry. While we were open to the responses of non-Palestinian health workers who work in the system, their experience or its possible difference from the median experience is not our principal focus. Between 91 and 100% of respondents are Palestinian, but because a few participants may not be we say “health workers in Gaza,” in our aims, consistent with our inclusion criteria.”

**Action** **taken:** none

Background:

2. Overall, the background section needs to be more streamlined toward the objective of the study which is also not clearly outlined. What is the objective, what are the research questions, and what background information is relevant to understand the results?

**Authors’ Response:** Thank you for feedback focus on the introduction. We re-wrote the final paragraph of the introduction to the background section so that the answers to these questions become clear more immediately.

**Action taken:** The rewritten text is highlighted below.

**Relevant text (lines 84-91)**: “The targeting of the healthcare system and health workers in Gaza has been a particularly egregious feature of the recent assault. The aim of our study is to learn about the violations suffered by health workers in Gaza during this period, as measured by their loss of loved ones and colleagues and their workplace displacement, and its psychological consequences, and measured by the ProQOL-H, which is described in detail below. To interpret the data in the context of genocide, we draw on literature on severe trauma and its consequences, existing literature on culturally specific strategies for coping with trauma in Palestine, and the broader literature on the well-being of health workers and its importance for health system functioning.”

Intro of background section:

3. The background information is as such good, but it is difficult to know how it relates to the study objective. Is there any way that the authors can lead with their objective and then outline the context in which the study is carried out – one of illegal occupation, apartheid, and genocide. Also, in relation to the genocide, the health-related consequences could have been highlighted in a more integrated way. At this point, the readers doesn’t learn anything about the disastrous health situation that physicians and nurses are dealing with. I find this an oversight.

It would have been useful to end the section with a clear objective and research questions that guide the piece (I am not convinced that the piece is making a theoretical contribution; it is making an important empirical one).

**Authors’ Response:** We agree that the principal contribution we are making is empirical, not theoretical. However, we drew on an extensive body of relevant literature in order to properly interpret our data. The final paragraph of the introduction to the background section has been rewritten to make this clear.

**Action taken:** The rewritten text is highlighted below.

**Relevant text (lines 84-91)**: “The targeting of the healthcare system and health workers in Gaza has been a particularly egregious feature of the recent assault. The aim of our study is to learn about the violations suffered by health workers in Gaza during this period, as measured by their loss of loved ones and colleagues and their workplace displacement, and its psychological consequences, and measured by the ProQOL-H, which is described in detail below. To interpret the data in the context of genocide, we draw on literature on severe trauma and its consequences, existing literature on culturally specific strategies for coping with trauma in Palestine, and the broader literature on the well-being of health workers and its importance for health system functioning.”

Targeting of Health Workers and Health Infrastructure in Gaza:

4. P7: The text would benefit from a more structured flow. It currently jumps between affected people, infrastructure, and patterns of attack. I suggest starting with the impact on people, then addressing infrastructure, and finally discussing attack patterns—without shifting back and forth between these topics.

**Author’s response:** We appreciate this feedback. The purpose of this paragraph is not to systematically describe all of the many kinds of attacks on healthcare, but to briefly characterize its severity and introduce the idea that its psychological effects are likely also unprecedented and in need of study.

**Action taken:** We adjusted the title of this section to better describe its purpose.

**Relevant text (line 92):** “Targeted Attacks on Healthcare in Gaza and Their Impact on Health Workers.”

Psychological Constructs for Health Worker Distress and Well-being:

5. I would have moved this section following the section ‘Studying the Psychological Effects of Genocide’ – like this it interrupts the flow from understanding how the horrendous situation in Gaza affects health workers. This can then be followed by a section about how the international literature has tried to make sense of this more generally by introducing the various relevant concepts.

**Author’s response:** The development of a ProQOL-H took place outside the context of Palestine, and outside a context of targeted violence against health workers, and so this needs its own section since we rely heavily on this instrument. We prefer this structure so that we can emphasize the unique experiences of health workers and the tools used to study them, and the context of this genocide in which health workers have not been protected as is usually expected.

**Action taken:** We added a transition sentence to better connect the section on psychological constructs with the final background section on genocide, highlighted below.

**Relevant text (lines 160-167):** “While existing literature acts as a guide to the study of mental health in Palestine generally and of health workers in Palestine, the current situation for health workers in Palestine is uniquely horrific. For those who survive, they continue to live in perpetual proximity to death.(41) The mental health of health workers is important to the functioning of any health system, but the psychological toll of the ongoing genocide must be studied and understood within its violent political context and the targeting of health workers. While the study of nurses in the West Bank in 2024 reflected an average professional quality of life, the brutal treatment of health workers during the genocide in Gaza needs additional attention.

Studying the Psychological Effects of Genocide:

6. The section needs to be more focused on the psychological effects of genocide among healthcare workers. The destruction of the social and cultural fabric could have been mentioned in the background above rather than here. That is, after a section that outlined the targeting of healthcare workers, I would have expected a focus on the evidenced and/or expected psychological health consequences. The authors could have engaged with health reports to explore how health workers express their distress.

**Author’s response:** Thank you for the thorough attention paid to the structure of the background section. As mentioned above, we feel that narrowing the focus of this section on health workers or integrating details of the technical literature behind ProQOL-H into this section could take away the particular attention needed for the study of genocide. The limitation of existing literature is the fact that the study of health worker mental health is not integrated with the study of genocide.

**Action taken:** None. The closing paragraph of the background section summarizes the need to draw on multiple disparate bodies of literature to understand the current situation for health workers in Palestine:

**Relevant text (lines 190-197):** “We use the word “genocide” to refer to this period of accelerated violence and displacement in Gaza, following preliminary ICJ findings and subsequent detailed reporting by UN Special Rapporteur Albanese.(4,5) It is also consistent with more recent reports of the United Nations Special Committee to Investigate Israeli Practices,(49) Human Rights Watch,(50) and Amnesty International(51) all finding that Israel has indeed committed genocide in Gaza. The psychological consequences for health staff enduring this can be understood in relation to past work on the mental health of Palestinian health workers, the mental health of other victims of genocide,(46) and the broader literature on the well-being of health workers. “

Methods:

7. Thank you for improving this section

Results:

Results are presented clearly and are informative.

8. A stylistic note: P16/line292: “Additionally, 41 health workers…” using ‘additionally’ makes the experiences of personal loss sound slightly like an afterthought. I would say: “Personal losses were experienced by 41 health workers due to Israeli military violence (Table 3)…”

**Author’s response:** Thank you for this suggestion.

**Action taken:** We have implemented the change as suggested.

**Relevant text (lines 294-295): “**Personal losses were experienced by 41 health workers as a result of Israeli military violence (Table 3).”

Discussion:

The discussion summarises the key findings well.

9. I still struggle somewhat with the extrapolation from the literature on idioms of distress and steadfastness to explain perceived support and levels of moral distress. One aspect that seems overlooked, for example, is the role of adrenaline in keeping people going under even the most horrendous circumstances. If the authors wish to keep the text as it stands, it would benefit from further streamlining and clarification how experiences of trauma, mental distress, and steadfastness might affect the ways in which health workers regarded their level of distress and support received.

**Author’s response:** Thank you for these comments. We refer to literature on idioms of distress and steadfastness because we believe they may be the best analogous concepts in existing literature, but we emphasize that this must be done with caution. While adrenaline and the stress response are relevant to the very broad literature on stress and health, we do not have any basis for claiming that it has special relevance here.

**Action taken:** To further emphasize the limitations regarding the moral distress and perceived support data we added the sentence highlighted below.

**Relevant text (lines 378-384):** “These findings and background support the two conceptions of *sumud* by Wick and Meari – the structured capacity to cope with oppression is also a foundation for anti-colonial resistance.(27,28) Still, we must interpret these data with caution. Because perceived support and moral distress were not included in the version of ProQOL used by Ayed et al, the interpretation of these two measures does not benefit from comparison to another sample of Palestinian health workers. Interviews and qualitative analysis would be needed to understand if there is meaningful overlap between ProlQOL-H constructs and *sumud* or other culturally-specific responses to hardship.”
