# Supplementary material for: Displacement, personal loss, and psychological strain among physicians and nurses working in Gaza, 2023–2024
Source: PLOS Glob Public Health. 2025 Sep 10;5(9):e0005094. doi: 10.1371/journal.pgph.0005094 (PMC12422511; doi:10.1371/journal.pgph.0005094)
Supplement: Attachment — Submitted filename: 20250721 Response to Reviewers.docx [file pgph.0005094.s009.docx]

To the editors of PLOS Global Public Health,

Thank you for the opportunity for the opportunity to further revise our manuscript. Changes made in response to your requested edits and recommendations are below:

**Comment 1: “**Regarding the title, PLOS has found that general and social media coverage tends to include the title of articles only; researchers often read only title and abstract; as such, the important context and explanation around use of the term ‘genocide’ provided in the body text will likely not be seen by most readers. The presumptive lack of that context and explanation is likely to trigger strong negative reactions (perhaps more in some media contexts than in others), including accusations of bias/partisanship. If accusations of bias/partisanship dominate the media coverage of/reader reaction to the article, the actual content of the article—and much of the important impact it could have—can likely be overshadowed.

Thus, a suggestion has been made regarding the following alternative phrases to be included in the title in place of the word "genocide":

Israel's war on Gaza
Israel's onslaught on Gaza
Israel's assault on Gaza
Israel's ongoing offensive in Gaza
Israel's siege in Gaza”

**Authors’ Response:** We have simplified the title to “Displacement, Personal Loss, and Psychological Strain Among Physicians and Nurses Working in Gaza, 2023-2024"

**Comment 2**: “We'd also recommend that you add a sentence to the abstract that summarizes this excerpt from the introduction: ‘“We use the word “genocide” to refer to this period of accelerated violence and displacement in Gaza, following preliminary ICJ findings and subsequent detailed reporting by UN Special Rapporteur Albanese.(4,5) It is also consistent with more recent reports of the United Nations Special Committee…’ so as to provide additional context in the abstract itself. This is because readers and the media often only read the title and the abstract of research papers and might miss the careful justification you include around the choice of this framework.”

**Author’s Response:** Thank you for this prudent recommendation. We have added language below. Because of growing consensus among genocide scholars about the situation in Gaza, we have also mentioned them.

**Relevant text:**

**Lines 42-45:** “We use the word “genocide” to refer to this period of accelerated violence and displacement in Gaza, following preliminary ICJ findings, subsequent detailed reports by multiple human rights organizations, and statements by genocide scholars concluding that Israel is indeed committing genocide in Gaza.”

**Lines 193-198:** “We use the word “genocide” to refer to this period of accelerated violence and displacement in Gaza, following preliminary ICJ findings and subsequent detailed reporting by UN Special Rapporteur Albanese.(4,5) It is also consistent with more recent reports of the United Nations Special Committee to Investigate Israeli Practices,(49) Human Rights Watch,(50) and Amnesty International(51) all finding that Israel has indeed committed genocide in Gaza. These finds are echoed increasingly by genocide scholars. (52)”

**Comment 3:** “Finally, we recommend that you cite a recent peer-reviewed paper that uses genocide as framework as a precedent to further substantiate the concepts they use (Hamamra, B., Mahamid, F., Bdier, D., & Atiya, M. (2025). War-related trauma in narratives of Gazans: challenges, difficulties and survival coping. Global mental health (Cambridge, England), 12, e34. <https://doi.org/10.1017/gmh.2025.23>)”

**Author response:** Thank you for this recommendation. We found this paper after the previous round of revisions and planned to reference it.

**Relevant text**

**Lines 421-424: “**Our study expands upon empirical work on the genocide by Hamamra et al, who conducted a qualitative study of trauma narratives and coping among 30 displaced Palestinian civilians in Rafah, interpreting their results through the lens of genocide as a distinct traumatic exposure. (63)”
