# Supplementary material for: Displacement, personal loss, and psychological strain among physicians and nurses working in Gaza, 2023–2024
Source: PLOS Glob Public Health. 2025 Sep 10;5(9):e0005094. doi: 10.1371/journal.pgph.0005094 (PMC12422511; doi:10.1371/journal.pgph.0005094)
Supplement: Attachment — Submitted filename: Reviewer comments.docx [file pgph.0005094.s005.docx]

PGPH-D-24-02208

Displacement, Personal Loss, and Psychological Strain Among Physicians and Nurses in Gaza during Israel’s Genocide in Palestine, 2023-2024

Abstract:

The abstract is well written and clear. I would suggest to also refer to the ICJ and its claim that what we are witnessing is genocide.

Main document:

It would be helpful to start with a brief introduction that outlines the objective of the study, justifies its importance, outlines the research gap, and lists the research questions with which the authors seek to fill the gap. An outline of the article and how it contributes to research and practice would also be helpful. This would help the reader to understand the relevance of the following sections.

Background:

Overall, the background section could be better structured. Right now it is not always easy to follow. My suggestion is:

(a) Background on the current genocidal war on Gaza and connected to this the number of people killed and injured, neighbourhoods flattened, and vital infrastructure destroyed. (Here you can refer immediately to Albanese’s report and the ICJ and other documentation)

(b) Focus on the health system (what used to be there); the almost total destruction of the health infrastructure; and the conditions under which health professionals have to work

(c) The impact of working under such conditions (while living through the genocide themselves) on health workers’ health and mental health as far as we know it

(d) Contextualisation of their experiences in relation of previous onslaughts and what we know about their health and mental health during these. The expected cumulative effect can be mentioned which brings it back to the present.

(e) Final note of why understanding health workers mental health is so important

Other comments on background:

It would be important to state that the health system has been completely destroyed and brough to its knees. More information about the destruction of infrastructure and the difficulty of accessing medication and other vital supplies would provide important contextual insight to better understand the almost impossible situation in which health workers need to carry out their work.

The psychological strain among health providers needs to be understood in the wider context of siege, war, and violence. Would it be useful to also refer to other recent onslaughts in 2008/2009, 2012, 2014, and 2021 and then of course the Great March of Return in 2018 (you mention the latter) which caused many injuries and disabilities especially among young people. Health professionals will have seen horrific injuries and other war related health problems before the genocide which leave their psychological marks.

Methods:

The methods section seems to suggest that you didn’t only include Palestinian health workers, but anyone who worked/volunteered in the Gaza health sector before and sometime after October 7^th^. I think this needs to be made much clearer from the start (i.e., objective and research questions (which are absent). It would also need to feature in the background more strongly. The background needs to highlight that Gaza’s health system is supported by international health workers/volunteers with relevant numerical and demographical information provided.

Results:

These are important and well reported.

While you moved qualitative quotes in the appendix as there were too few for thematic analysis, I wonder if they could be included in relevant sections as illustrative examples of how participants reflected on what the numbers reveal. You can note the lack of methodological rigour here in the methods section – I just find that these quotes bring the numbers to life and form part of your findings.

Discussion:

Much of the information here reads like it belongs in the background section. It would have been useful for readers to read about the destruction of hospitals and the mass graves that were uncovered in them beforehand.

While the reference to sumud is apt, it did not come up in the survey. Conflating sumud with compassion might not be entirely appropriate. It would have been good for the survey to reflect more locally relevant expressions of distress and strengths. This, in my view, is another key limitation: i.e., Western conceptions were inquired into while the interpretation tries to make them locally/culturally relevant without having the necessary data. Considering this, the section on sumud is too detailed and long. Instead, it would have been useful to know how these findings reflect experiences of health workers in other war affected settings. What is particular about this situation, what similarities exist?

Finally, I would have liked to know what practical lessons the research inspires. What should be done to support health providers in the short-, medium-, and long-term?
